# From sink to source: high inter-annual variability in the carbon budget of a Southern African wetland

**DOI:** 10.1098/rsta.2021.0148

**Published:** 2022-01-24

**Authors:** Carole Helfter, Mangaliso Gondwe, Michael Murray-Hudson, Anastacia Makati, Ute Skiba

**Affiliations:** ^1^ UK Centre for Ecology and Hydrology, Atmospheric Chemistry and Effects, Penicuik EH26 0QB, UK; ^2^ Okavango Research Institute, University of Botswana, Maun, Botswana

**Keywords:** carbon dioxide, methane, tropical wetland, drought, Africa, Okavango Delta

## Abstract

We report on three years of continuous monitoring of carbon dioxide (CO_2_) and methane (CH_4_) emissions in two contrasting wetland areas of the Okavango Delta, Botswana: a perennial swamp and a seasonal floodplain. The hydrographic zones of the Okavango Delta possess distinct attributes (e.g. vegetation zonation, hydrology) which dictate their respective greenhouse gas (GHG) temporal emission patterns and magnitude. The perennial swamp was a net source of carbon (expressed in CO_2_-eq units), while the seasonal swamp was a sink in 2018. Despite differences in vegetation types and lifecycles, the net CO_2_ uptake was comparable at the two sites studied in 2018/2020 (−894.2 ± 127.4 g m^−2^ yr^−1^ at the perennial swamp, average of the 2018 and 2020 budgets, and −1024.5 ± 134.7 g m^−2^ yr^−1^ at the seasonal floodplain). The annual budgets of CH_4_ were however a factor of three larger at the permanent swamp in 2018 compared to the seasonal floodplain. Both ecosystems were sensitive to drought, which switched these sinks of atmospheric CO_2_ into sources in 2019. This phenomenon was particularly strong at the seasonal floodplain (net annual loss of CO_2_ of 1572.4 ± 158.1 g m^−2^), due to a sharp decrease in gross primary productivity. Similarly, drought caused CH_4_ emissions at the seasonal floodplain to decrease by a factor of 4 in 2019 compared to the previous year, but emissions from the perennial swamp were unaffected. Our study demonstrates that complex and divergent processes can coexist within the same landscape, and that meteorological anomalies can significantly perturb the balance of the individual terms of the GHG budget. Seasonal floodplains are particularly sensitive to drought, which exacerbate carbon losses to the atmosphere, and it is crucial to improve our understanding of the role played by such wetlands in order to better forecast how their emissions might evolve in a changing climate. Studying such hydro-ecosystems, particularly in the data-poor tropics, and how natural stressors such as drought affect them, can also inform on the potential impacts of man-made perturbations (e.g. construction of hydro-electric dams) and how these might be mitigated. Given the contrasting effects of drought on the CO_2_ and CH_4_ flux terms, it is crucial to evaluate an ecosystem's complete carbon budget instead of treating these GHGs in isolation.

This article is part of a discussion meeting issue ‘Rising methane: is warming feeding warming? (part 2)’.

## 1. Introduction

Concentrations of atmospheric methane (CH_4_), the second most important GHG after carbon dioxide (CO_2_) [1–3], have increased steadily since 2007 after nearly a decade of stability [4–7], with an annual growth rate in 2017 of 6.1 ± 1.0 ppb yr^−1^, equivalent to 16.8 Tg yr^−1^ [8]. While the relatively short atmospheric lifetime of CH_4_ offers opportunities to rapidly reduce its warming influence [9,10], the causes for the renewed increase in atmospheric concentration are not yet fully understood. Possible explanations include an increase in CH_4_ emissions from anthropogenic sources such as oil and natural gas [11], a reduction in CH_4_ destruction due to changes in the oxidative capacity of the atmosphere [12,13] and an increase in biogenic emissions, due to e.g. climate anomalies [14]. Observations of a shift in the isotopic signatures of atmospheric CH_4_ support the idea of an increase in net emissions from microbial sources and identify tropical areas are substantial contributors [15,16], with 65% of the global CH_4_ budget attributed to latitudes less than 30° [8].

The growth rate of atmospheric CO_2_ has accelerated from 1.8 ± 0.07 Gt C yr^−1^ in the 1960s to 4.9 ± 0.02 Gt C yr^−1^ in 2009–2018 [17], but inter-annual variability is large. While anthropogenic emissions continue to rise [18], the fate of the ocean and land sinks continues to be debated: some studies concluded that factors such as reduced net primary productivity and increased ecosystem respiration [19–22] may weaken the strength of those CO_2_ sinks, while others report growth in uptake due to longer growing seasons [17,23].

There is a recognized need to improve the understanding of the biogenic processes and controls underpinning net uptake and emissions of carbon. Deriving better emission factors and refining the mapping of wetlands and inundated soils will be particularly important to improve the representation of wetlands and inland waters in process-based models and GHG inventories and prevent double accounting [24]. Carbon dioxide and methane cycles in wetlands are complex: net fluxes between surface and atmosphere result from concomitant and competing processes such as microbial CH_4_ production and oxidation in the soil, and CO_2_ drawdown through photosynthesis and loss through vegetative respiration. Wetland hydrology is a key control of CO_2_ and CH_4_ fluxes, but the effects of fluctuating water table on these terms can be unpredictable: for example, ecosystem respiration has been reported to be insensitive to water table, or increase or decrease with water table, depending on what its dominant controls are [25,26]. Estimates put the proportion of the global wetland carbon pool contained in tropical wetland soils between 14% and 19%, but current understanding of how environmental conditions and their fluctuations impact CO_2_ and CH_4_ fluxes is limited [27,28]. The knowledge gap is particularly large in tropical areas due to the paucity of measurement capacity. The tropics make a disproportionate contribution to the global CH_4_ and CO_2_ budgets (e.g. 2/3 of global anthropogenic and biogenic emissions of CH_4_ [8]), and it is hence crucial to expand the observation network for CO_2_ and CH_4_ in both time and space to provide better coverage in this broad climatic zone. Multi-year assessments using an array of measurement approaches (e.g. ground-based and earth observation) are required to capture temporal changes in CO_2_ and CH_4_ fluxes in response to fluctuations in environmental conditions, resolve processes, and aid modelling and upscaling from local to regional and global scales.

In this paper, we present three years of continuous measurements of CO_2_ and CH_4_ fluxes by eddy-covariance in the Okavango Delta in Botswana, southern Africa. We studied the temporal variability of the fluxes at two hydrologically distinct sites, and compared seasonal and annual emission budgets upscaled to the entire Delta during two contrasting years. The aims of this study were to (i) establish, for the first time, CO_2_ and CH_4_ emission budgets for the Okavango Delta, (ii) identify their key environmental drivers and (iii) quantify the impact of drought, a major climatic stressor, on the wetland's carbon budget.

## 2. Methods

### (a) Study sites

The Okavango Delta is a large endorheic wetland complex in northern Botswana with a surface area of 40 000 km^2^. The climate of Botswana is classed as arid under the Köppen–Geiger classification scheme. Due to differences in aridity within the country, central and south-west areas are classed as BWh (main climate B—arid, precipitation W—desert and temperature h—hot arid), while northern and eastern areas fall into the BSh class (main climate B—arid, precipitation S—steppe and temperature h—hot arid) [29]. The Okavango Delta is fed by the Okavango River and receives pulsed flooding of 8.5 ± 2.0 Gm^3^ yr^−1^ (mean and standard deviation of annual cumulative discharge at Mohembo for the period 1975–2020; 70% of the annual inflow) through river discharge originating in the Angolan Highlands. During the rainy season (typically October–March) an additional 3.8 ± 1.2 Gm^3^ yr^−1^, 30% of the annual inflow, is received as rainfall [30]. The floodwaters travel from the main inlet at Mohembo to the main outlet in Maun (250 km as the crow flies) in four to five months. Peak flood typically occurs in August but the maximum annual extent depends on the combined inputs from floodwater and precipitation, which can vary from year to year [31–34]. As a result of this, three hydrographic zones can be defined: (i) perennial swamps, (ii) seasonal swamps, flooded three to six months per year and (iii) occasional swamps, flooded once per decade or less frequently. These flooding regimes are responsible for a marked zonation of the vegetation within the Okavango Delta: perennial swamps are dominated by reed grasses and sedges such as *Phragmites* spp. and *Cyperus papyrus*; these also occur along the channels which meander through the seasonal floodplains, where species such as *Panicum repens* and *Oryza longistaminata* are conspicuous. In contrast, non-aquatic vegetation, trees, shrubs and areas of bare, salt-crusted soil are typically found in the sparsely vegetated interior of many of the Delta's islands.

**Figure 1.**
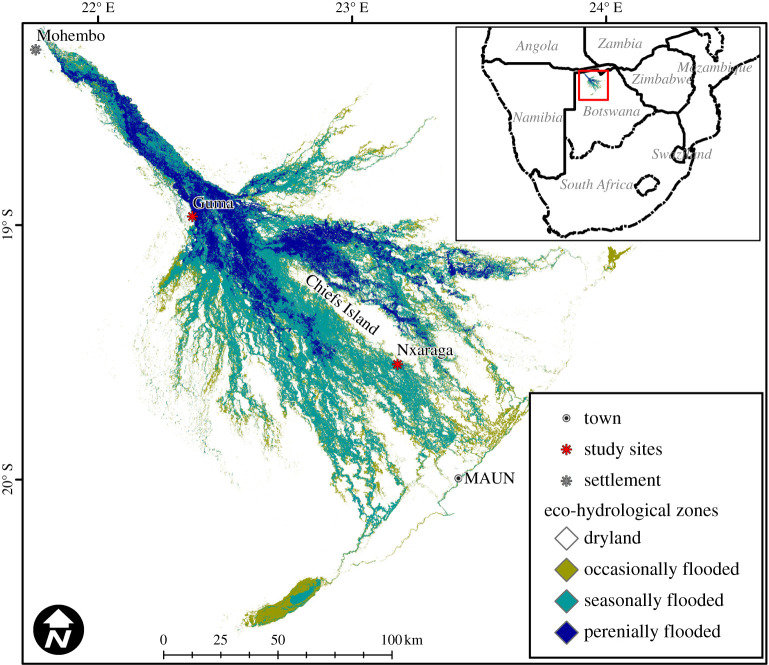
Eco-hydrological zones of the Okavango Delta in 2019, based on a 25-year flood record and frequency-determined floodplain vegetation communities [35,36]. (Online version in colour.)

We established two eddy-covariance (EC) measurement sites in the Okavango Delta: in the perennial swamp at Guma Lagoon (18°57′53.01′′ S; 22°22′16.20′′ E), and in the seasonal swamp, at Nxaraga (19°32′53′′ S; 23°10′45′′ E) on the S edge of Chief's Island (figure 1).

### (b) Instrumentation

The main components of the EC system were a Campbell Scientific IRGASON (three-dimensional ultrasonic anemometer and open-path infrared gas analyser, providing co-located measurements of the wind vector and mass densities of CO_2_ and water vapour) and a LI-COR 7700 open-path CH_4_ analyser. The anemometer was oriented into the prevailing wind. The vertical separation between the anemometer and the CH_4_ analyser was approximately 0.0 m (i.e. middle points of the sonic and optical paths in the same horizontal plane) and the lateral separation, in the crosswind plane, was approximately 0.3 m. Standard meteorological variables were measured: air temperature, pressure, relative humidity, wind speed and wind direction (Vaisala WXT520 weather station), total solar radiation (Skye Instruments pyranometer, model SKS1110) and photosynthetically active radiation (PAR; quantum detector, model SKP215). The data were logged using a Campbell Scientific CR3000 datalogger (10 Hz for the EC variables and 10-s interval for the meteorological parameters). Details of flux calculations and data quality control are provided in the electronic supplementary material.

At Guma Lagoon (18°57′53.01′′ S; 22°22′16.20′′ E), the EC system was installed on land, 30 m from a predominantly floating papyrus (*C. papyrus*) mat. The average papyrus canopy height was of the order of 2.5 m above water level and the effective measurement height of the EC system was 5.5 m. The papyrus plants at the shoreline were rooted and the mat extended *ca* 300 m into the lagoon in an easterly direction. The maximum depth of the lagoon varies between 2 and 6 m, depending on inflow, and the surface area is approximately 0.97 km^2^. A narrow margin (approx. 3 m) of floating-leaved macrophytes (mainly *Nymphaea* spp.) populate *ca* 50% (3.5 km) of the perimeter of the open water, which represents a surface area of 0.01 km^2^.

The EC system at Nxaraga (19°32′53′′ S; 23°10′45′′ E) was installed atop a 2.5-m high tripod erected on the southern edge of Chief's Island. The EC system overlooked a seasonal floodplain, which extends several hundred metres to the SE to SW. A permanent water channel fringed by reeds and grasses such as *Phragmites* spp. and *Miscanthus junceus* meanders through the floodplain, whose vegetation is dominated by grass species such as, *P. repens*, *Cynodon dactylon* and *Sporobolus spicatus*. The floodplain is grazed for most of the year by a variety of herbivores (e.g. impala, buffalo, elephant, hippopotamus), whose movements churn the soil, particularly in riparian areas.

### (c) Monthly and annual budgets

The half-hourly data were aggregated into hourly bins to construct 24-h mean time series of CO_2_ and CH_4_ fluxes for each month of the study (August 2017–April 2021). The uncertainty associated with each hourly mean data point was set to the ensemble standard deviation calculated for all data points averaged into each hourly bin. This data aggregation approach ensured equal weighting for each of the 24 hourly points of the monthly. Since night-time fluxes are more likely to fail the data quality control tests, aggregating into diel cycles reduces the risk of biasing higher temporal statistics (e.g. daily, monthly or annual budgets) towards daytime values. Aggregating into hourly rather than half-hourly values increased the number of points available in each bin and improved the statistics of the calculated fluxes.

Hourly values were summed to produce the daily budgets and the associated uncertainties (*σ*_day_) were calculated using standard error propagation rules (equation (2.1); *σ_i_* denotes the uncertainty on the flux value at hour *i*, i.e. ensemble standard deviation described above).

2.1 $$\sigma_{\text{day}}=\sqrt{\overset{23}{\underset{i=0}{\sum }}{\left(\sigma_{i}\right)}^{2}}.$$

Monthly budgets and uncertainties were calculated by multiplying the daily values by 365/12 (i.e. all monthly values are directly comparable because they have the same number of days) and annual budgets were obtained by summing the monthly values. Following error propagation rules, monthly uncertainties were summed in quadrature as in equation (2.1) to obtain the total annual uncertainty.

### (d) Eco-hydrological zones of the Okavango Delta

Satellite imagery, aerial photography, ground-truthing, rule-based modelling and combinations thereof have been used to map the extent and types of flood zones in the Okavango Delta [37–40]. Because these published zone maps are dated and of relatively low spatial resolution, and because flooding is dynamic and variable, we produced, for this study, a new distribution map of eco-hydrological zones using a recent time series of higher resolution remote sensing (figure 1) and statistically determined plant communities [35]. We aggregated the plant communities into larger groups for this study, in order to distinguish between perennially flooded areas and seasonal floodplains.

The annual flood frequency in the Okavango Delta was mapped by layer-stacking a dataset of existing, publically available, maximum inundation extent maps derived from Landsat imagery [36] spanning the period 1990–2019. This dataset excludes the five years (1993, 2000, 2009, 2010 and 2012) within this time frame, for which one or more of the six annual images needed to produce the mosaicked composite image for the year were not available. Flood frequency transition thresholds were determined from macrophyte species distribution modelling, cluster analysis and indicator species analysis in earlier work [35]. These were used to define boundaries between eco-hydrological zones characterized by specific species assemblages (figure 1). In 2019, the inflow was the lowest on record (1934–present) and caused a contraction of both the perennially and seasonally flooded areas that year, effectively increasing the extent of the occasionally flooded area. To capture the 2019 shift, the annual extent of the perennially flooded area was subtracted from the total inundated area derived from high temporal resolution MODIS imagery [38,39].

### (e) Flux upscaling

A simple upscaling approach was used to calculate the annual budgets of CO_2_ and CH_4_ for the entire Okavango Delta using the temporally and spatially weighted monthly budgets obtained at the EC measurement sites in the permanent and seasonal swamps and the distribution of the eco-hydrological zones of the Delta (equation (2.2)). The total annual budgets were calculated as the sum of the individual budgets of the perennial, seasonal and occasional wetlands. These terms were calculated by summing the monthly budgets (§2c) and upscaling them to the area covered by each eco-hydrological zone (§2.4, table 2). The annual budgets for CO_2_ and CH_4_ over dry, sandy soil were estimated from static chamber measurements at Nxaraga [41]. These measurements were taken monthly on Chief's Island from February 2018 until August 2020 (no data for May–June and December 2018, January 2019, April 2019, June–August 2019 and January–June 2020) *ca* 10–20 m inland, in a northerly direction from the EC mast overlooking the seasonal floodplain, and were used as proxies for land-atmosphere exchange of CO_2_ and CH_4_ in occasionally flooded areas of the Delta.

2.2 $$F_{\text{DELTA}}=\overset{12}{\underset{i=1}{\sum }}\{A_{P}F_{P,i}+A_{S}F_{S,i}+A_{O}F_{O,i}\}.$$

In equation (2.2), the surface areas of the permanent, seasonal and occasional wetlands (table 2) are denoted *A_P_*, *A_S_* and *A_O_*, respectively. *F_P,i_*, *F_S,i_* and *F_O,i_* denote the mean CH_4_ flux during month number *i* in the permanent, seasonal and occasional wetland, respectively.

## 3. Results

During the study period (August 2017–April 2021) the mean annual temperature was 26.8 ± 3.4°C (minimum 14.5 ± 4.1°C and maximum 35.1 ± 3.7°C) at Guma and 25.3 ± 2.6°C (minimum 13.7 ± 5.3°C and maximum 34.8 ± 3.2°C) at Nxaraga. July was the coldest month of the year (20.7 ± 4.8°C and 21.0 ± 6.0°C at Guma and Nxaraga, respectively) and October the hottest (28.7 ± 6.9°C and 28.1 ± 6.3°C at Guma and Nxaraga, respectively).

**Figure 2.**
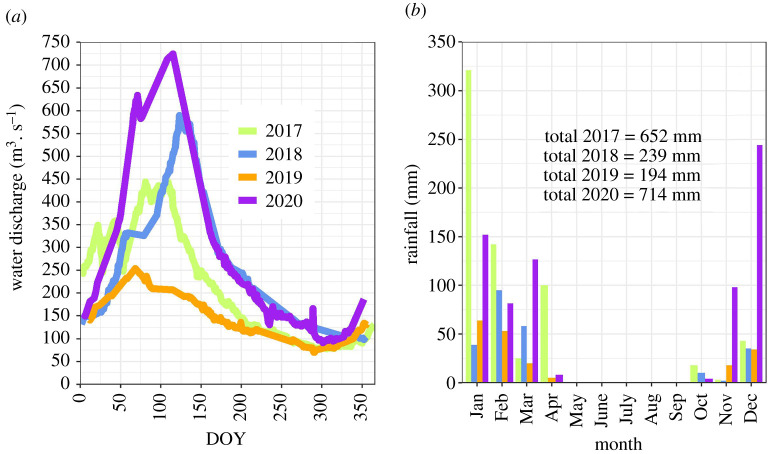
Monthly (*a*) mean water discharge measured at Mohembo (main inlet into the Okavango Delta), and (*b*) rainfall measured at Guma Lagoon. (Online version in colour.)

Relative humidity exhibited pronounced seasonality, and ranged from *ca* 20% in winter to 65% in summer. Global solar radiation was high all year round. Daily values ranged on average from 18 MJ m^−2^ in June to 26 MJ m^−2^ in December and were consistent with published data for the region [42]. The maximum water level in Guma Lagoon (arbitrarily measured off a deck on stilts *ca* 200 m north of the EC mast) varied almost two-fold during the study period, with peak heights of 0.69 m and 1.24 m measured in 2019 and 2020, respectively. Maximum flooding at Guma in 2019 occurred in mid- to late April, and early to mid-May in 2020. This time lag is consistent with differences in the timing of peak water discharge (early March 2019 and late April 2020) measured at Mohembo (inlet into the Okavango Panhandle; figure 2*a*). Furthermore, 2019 had the lowest peak water discharge (254 m^3^ s^−1^ in March 2019), and the lowest annual cumulative discharge (4.6 ± 0.1 Gm^3^, *ca* half the 1975–2020 mean value) since records began in 1975. Annual rainfall, measured at Guma Lagoon, was variable and ranged from 194 mm in 2019 to 714 mm in 2020. Precipitation occurs typically between October and April (figure 2*b*). Peak flood at Guma Lagoon occurs typically 1–1.5 months after peak discharge is recorded at Mohembo; at Nxaraga, the time delay is of the order of 2–2.5 months.

**Figure 3.**
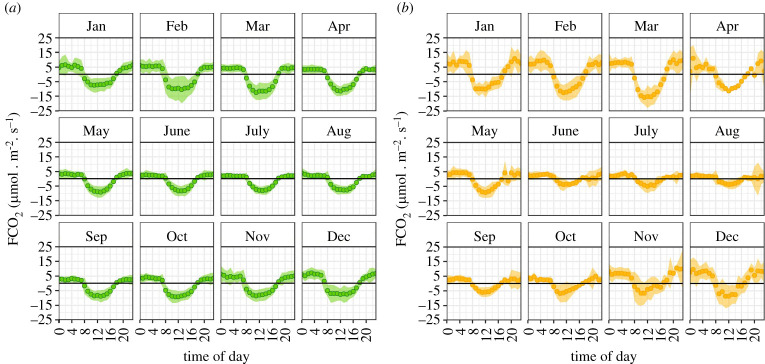
Diurnal trends in CO_2_ fluxes by month of the year measured over (*a*) a floating *Cyperus papyrus* mat at Guma Lagoon (perennial swamp) and (*b*) at Nxaraga seasonal floodplain. Circles represent hourly averages and ribbons the standard deviation (data range August 2017–April 2021). (Online version in colour.)

Carbon dioxide fluxes exhibited marked diurnal cycles at both the seasonal and perennial swamps with net uptake (negative flux values) by the vegetation during daylight hours (typically 8.00–18.00). The fluxes (both the net uptake during the day and net emissions at night) were smallest during the austral winter months (June–August) and largest in January–March. On average, the fluxes of CO_2_ were comparable in magnitude at the seasonal and perennial swamps, despite differences in vegetation and hydrological regimes (figure 3).

**Figure 4.**
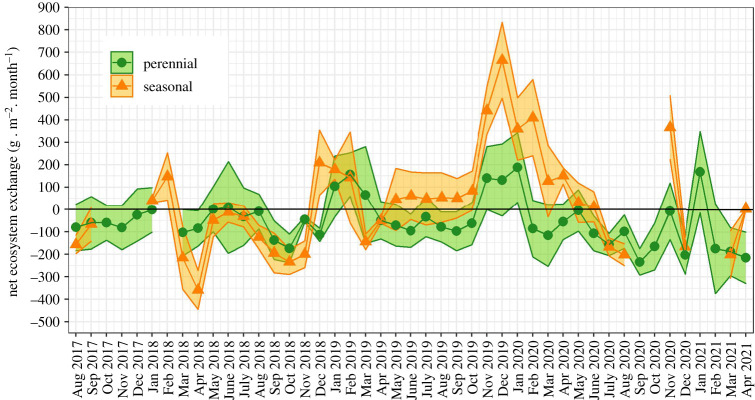
Monthly budgets and associated uncertainties (circles and ribbon) of net ecosystem exchange of CO_2_ measured at the perennial (Guma Lagoon) and seasonal (Nxaraga) swamps between August 2017 and April 2021. (Online version in colour.)

The seasonal trends in net ecosystem exchange (NEE) of CO_2_ were complex and exhibited high inter-annual variability (figure 4). In 2018, there was a marked bimodal trend with local maxima in net uptake in February–March and September–October. NEE was close to zero from *ca* May to July–August, and the fluxes were comparable between sites within uncertainty limits. The periods of net loss of CO_2_ to the atmosphere, typically observed from November to February, are noteworthy; this was particularly strong in 2019 for the seasonal floodplain ecosystem, where a maximum net loss of 665 ± 169 g m^−2^ month^−1^ was recorded in December 2019. Monthly fluxes at the seasonal floodplain were positive (loss to the atmosphere) throughout 2019, with the exception of March and April. These differences were compounded in the annual CO_2_ budgets which showed high variability between years and sites (table 1): the seasonal floodplain switched from being a strong sink in 2018 (−1024.5 ± 134.7 g m^−2^) to a strong source in 2019 (1572.4 ± 158.1 g m^−2^). The switch from net sink in 2018 (−742.0 ± 83.6 g m^−2^) to likely net source in 2019 (109.1 ± 121.2 g m^−2^) was also observed at the perennial swamp. In 2020, the papyrus at the perennial swamp switched back to being a net sink of atmospheric CO_2_ (−1046.4 ± 96.2 g m^−2^), and this annual budget was statistically different from 2019.

**Table 1.** Annual budgets of CO_2_ and CH_4_, carbon (C) and radiative balance measured at the perennial and seasonal swamps*.* The fluxes of CH_4_ were converted into CO_2_-eq units using a global warming potential of 28.

	Guma Lagoon (papyrus)				Nxaraga seasonal floodplain			
	FCH_4_ (g m^−2^)	FCO_2_ (g m^−2^)	C budget (g C m^−2^)	radiative balance (CO_2_-eq g m^−2^)	FCH_4_ (g m^−2^)	FCO_2_ (g m^−2^)	C budget (g C m^−2^)	radiative balance (CO_2_-eq g m^−2^)
2018	115.9 ± 14.9	−742.0 ± 83.6	−115.4 ± 25.4	2503.2 ± 425.5	36.3 ± 2.8	−1024.5 ± 134.7	−252.2 ± 36.8	−8.1 ± 155.8
2019	122.1 ± 14.4	109.1 ± 121.2	121.3 ± 34.8	3527.9 ± 421.0	8.7 ± 3.2	1572.4 ± 158.1	435.4 ± 43.2	1816.0 ± 181.7
2020	—	−1046.4 ± 96.2	—	—	—	—	—	—

**Figure 5.**
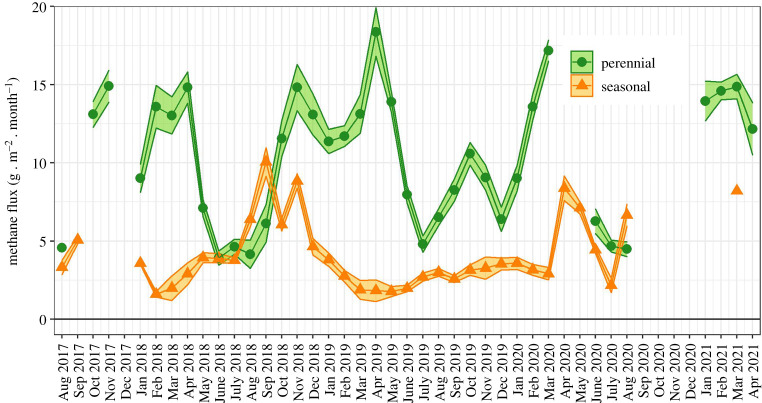
Monthly budgets and associated uncertainties (circles and ribbons) of CH_4_ fluxes measured at the perennial (Guma Lagoon) and seasonal (Nxaraga) swamps between August 2017 and April 2021. (Online version in colour.)

In contrast, CH_4_ emissions in the perennial swamp varied insignificantly between 2018 (115.9 ± 14.9 g m^−2^) and 2019 (122.1 ± 14.4 g m^−2^), but they decreased by a factor of four at the seasonal floodplain (36.3 ± 2.8 g m^−2^ in 2018, and 8.7 ± 3.2 g m^−2^ in 2019). The seasonal emission patterns differed between ecosystems too (figure 5): at the perennial swamp, we observed a bimodal CH_4_ signal characterized by minimum emissions in winter, comparable to the seasonal variations observed for CO_2_. The minimum CH_4_ emissions period was longer in 2018 (June–August) than in 2019 (June), but inter-annual differences in both temporal variability and flux magnitude were otherwise minimal. In contrast, at the seasonal floodplain, CH_4_ emissions were stable from January 2018 until April, and increased steadily before peaking at the end of the calendar year. The emissions were close to zero with negligible monthly variations from February 2019 until March 2020, when a sharp increase was recorded.

Both ecosystems switched from being net sinks of atmospheric carbon (C) in 2018 (−115.4 ± 25.4 g C m^−2^ at the perennial swamp and −252.2  ± 36.8 g C m^−2^ at the seasonal floodplain) to net sources during the 2019 drought year (121.3 ± 34.8 g C m^−2^ at the perennial swamp and 435.4 ± 43.2 g C m^−2^ at the seasonal floodplain, table 1).

Expressed in CO_2_-equivalent units using a global warming potential (GWP) value of 28, the CO_2_ uptake by the ecosystem of the seasonal floodplain exceeded the CH_4_ losses to the atmosphere in 2018, which means that this ecosystem's radiative balance was negative [43]. In contrast, the radiative balance of both ecosystems was positive in 2019 (table 1) and increased by comparable amounts between 2018 and 2019 at the two sites. Drought caused positive radiative forcing in 2019 in both ecosystems, and this was predominantly driven by a weakening of the CO_2_ sink strength.

**Figure 6.**
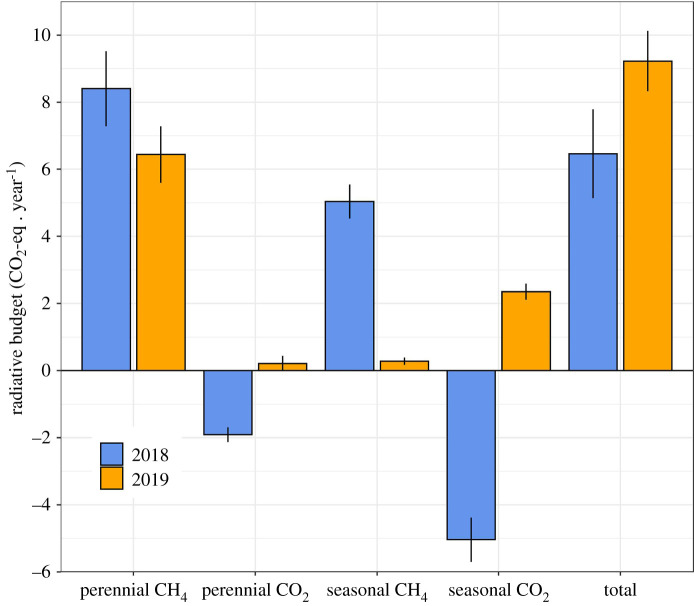
Annual budgets of CO_2_ and CH_4_ in 2018 and 2019 upscaled to the entire Okavango Delta, expressed in Tg CO_2_-eq yr^−1^. The total represents the radiative balance calculated as the sum of the individual contributions of the perennial and seasonal swamps. (Online version in colour.)

The extent of the seasonal and the perennial swamps decreased by 70% and 25%, respectively, between 2018 and 2019 (table 2). These changes, combined with the inter-annual variations in NEE of carbon (CO_2_ and CH_4_), resulted in a net increase of 43% in the radiative balance of the Okavango Delta as a whole between 2018 and 2019 (6.5 ± 1.3 Tg CO_2_-eq in 2018 to 9.2 ± 0.9 Tg CO_2_-eq in 2019; figure 6).

**Table 2.** Annual extent of the three main eco-hydrological zones in the Okavango Delta*.*

year	perennial (km^2^)	seasonal (km^2^)	occasional (km^2^)
2018	2575	4923	2243
2019	1911	1497	5669

## 4. Discussion

The large variability in the carbon budget of the Okavango Delta was driven by significant inter-annual changes in its hydrology. 2019 had the lowest water input into the Okavango Delta on record (total discharge at Mohembo 4.6 Gm^3^) and this was 40% smaller than in 2018 (figure 2*a*). This change in water influx is reflected in a change in the extent of the total flooded area (sum of perennial and seasonal wetlands), which is estimated to have shrunk by almost 50% between 2018 and 2019. This had a particularly adverse effect on the vegetation of the seasonal floodplains, which was decimated by desiccation (e.g. electronic supplementary material, figure S5) and fires. Ecosystem respiration (*R*_eco_) at the seasonal floodplain, evaluated from night-time fluxes of CO_2_, was an exponential function of air temperature in 2018; in contrast, *R*_eco_ was relatively low and constant from May to October 2019 compared to 2018, and not correlated with air temperature (electronic supplementary material, figure S6b), suggesting that *R*_eco_ was limited by water availability [44,45]. *R*_eco_ was lower in 2019 (4.1 ± 5.9 µmol m^−2^ s^−1^; median ± IQR) than in other years (6.4 ± 5.5 µmol m^−2^ s^−1^; median ± IQR), while GPP declined steadily from March until December 2019 (electronic supplementary material, figure S7b) as the drought intensified in the seasonal floodplain. This supports the idea that the switch from sink to source of CO_2_ between 2018 and 2019 was driven by a reduction in GPP, as a result of the drought-induced degradation of the vegetative CO_2_ sink, rather than fuelled by an increase in ecosystem respiration. The reduction in CH_4_ emissions between 2018 and 2019 at the seasonal floodplain was also consistent with drought; this finding was corroborated by chamber measurements within the flux footprint of the EC system, which established soil water content as a key driver for CH_4_ emissions [41], and it is also consistent with other published works which list water table or soil moisture as a major control [46–49]. Some studies have established GPP as a control of CH_4_ emissions at time scales as short as hours and days [25,50]; a reduction in GPP as a result of soil drying could have decreased the amount of C available to methanogens and, consequently, decreased CH_4_ production, but we did not find conclusive evidence of this (electronic supplementary material, figures S9b and S10b). We conclude that the inter-annual decrease in CH_4_ emissions at the seasonal floodplain is predominantly attributable to the effect of soil drying, which enables oxygenation of the soil making the edaphic environment less favourable to anaerobic processes such as methanogenesis.

The 2019 drought had an insignificant effect on the CH_4_ emissions from the papyrus ecosystem studied at Guma Lagoon; in contrast, the switch from sink to source of CO_2_ observed in this perennial wetland between 2018 and 2019 was predominantly driven by an increase in *R*_eco_ (+632 g m^−2^ yr^−1^, equivalent to 74% of the increase in NEE, and 62% of the total CO_2_-eq budget, in 2019). We attribute this added respiration term to a larger proportion of exposed sediments [49,51,52] and increased litter decomposition [53–55] as the water level in the lagoon receded in 2019. This is consistent with the findings of Jones *et al*. [56] who reported net losses of CO_2_ (306 g m^−2^ month^−1^) from papyrus during periods of hydrological drawdown, and net uptake (−489 g m^−2^ month^−1^) during inundated periods, at Lake Naivasha, Kenya. These values are at the high end of the measured fluxes in the Okavango Delta, which might be due to differences in climate (humid, hot tropical climate compared to semi-arid in Botswana), and in the degree of hydrological drawdown. Annual NEE of CO_2_ at Guma Lagoon (2018/2020 average of −894 g m^−2^ yr^−1^) was 50% smaller than at Lake Kirynia, Uganda (−1760 g m^−2^ yr^−1^, [57]).

As previously noted, the upscaled, Delta-wide budget is probably underestimated because the fate of emissions in areas that were not classified as perennially flooded in 2019 is uncertain. These areas would have implicitly been added to the extent of the seasonal floodplains without accounting for differences in vegetation type, abundance and without adjusting the associated GHG emission factors. In particular, soil CH_4_ emissions and ecosystem respiration in such re-classified perennial swamps are likely to have increased as the water levels dropped. Ascribing seasonal floodplain emissions to the areas which were not classified as perennial swamps during the 2019 drought year, could, therefore, have led to underestimations of the C and radiative budgets of these areas. In this light, the simple inter-annual budgets presented here are likely to be optimistic scenarios, and future work should focus on quantifying C fluxes from transitional ecosystems between seasonal and perennial wetlands.

Nevertheless, our findings highlight the complexity of the controls of ecosystem exchange of CO_2_ and CH_4_ in wetlands and the variability of their carbon budgets. With complex direct and indirect effects on the processes underpinning the net CO_2_ and CH_4_ fluxes measured, hydrology was the overarching control of inter-annual variability. Variable precipitation and river discharge have short-term effects on the hydrology of the Okavango Delta (wetland extent, flooding depth), and longer term, lagged impacts on vegetation composition [58,59]. The strongest predictors of seasonal floodplain vegetation composition in a given year are multi-decadal average antecedent flooding frequency, three-year antecedent duration, and years since the last flood [58]. This implies that considering the instantaneous (seasonal or annual) state of the wetland, such as wetland extent, a key modelling parameter for CH_4_ emissions [60] whose inter-annual variability has been shown to modulate surface-atmosphere CH_4_ and CO_2_ exchange globally [61–65], without accounting for the gradual impact on vegetation composition, distribution and abundance would likely result in misrepresenting changes to plant-mediated pathways and processes. Multi-year monitoring is hence required to quantify the legacy effects of drought on vegetation composition and GHG budgets and forecast how these might change in the longer term.

Wetland extent is a key parameter in CH_4_ emissions modelling [60]; it is estimated that human activities (e.g. agriculture and urbanization) have caused the loss of 33% to 54–57% of wetlands globally [66,67] and shorter-term variations linked to the El Niño Southern Oscillation (ENSO) have also been reported [64,65]. These short-term variations in wetland extent are thought to modulate CH_4_ and CO_2_ emissions, particularly in the tropics [61–63]. Differences in model outputs can however be large, both in terms of the estimates of the magnitude of inter-annual change in C fluxes and their sensitivities to environmental controls, which is unsurprising given the diverse range of drivers and their effects on C emissions observed experimentally. Efforts must be made to increase GHG monitoring in the tropics and generate the ground-truthing needed to better constrain models. The fate of the wetland vegetation (distribution, abundance, species succession), and by extension the fate of the associated C budget, are directly impacted by climate anomalies (variable precipitation patterns) and man-made disturbances (e.g. water abstraction, wetland draining), which affect wetland hydrology as observed in the Okavango Delta [35].

The sensitivity of the carbon sources and sinks to drought and the large inter-annual variations in flooding extent make such seasonally flooded ecosystems volatile in terms of their NEE of carbon, and this is likely to intensify as precipitation patterns change in future. Furthermore, as the current focus is on identifying and quantifying natural sources of CH_4_, particularly in the tropics, our findings exemplify the need to also consider CO_2_, which was responsible for 89% of the radiative forcing from the seasonal floodplains of the Okavango Delta as a result of drought (figure 6). This figure is likely to be an underestimate because we assumed that the extent of seasonal floodplains lost between 2018 and 2019 (reduction of 70%) was neutral with respect to CO_2_ exchange (we know from chamber measurements that this assumption is defensible for CH_4_ fluxes). Considering both CO_2_ and CH_4_ is hence crucial to understanding the complex effects of climatic variations and human modifications on an ecosystem's GHG budget. Treating CH_4_ in isolation would have led to the erroneous conclusion that the 2019 drought was beneficial from a GHG budget perspective, because it led to a 94% reduction in CH_4_ emissions; this, in turn, could have lent weight to controversial/emerging narratives advocating draining wetlands (e.g. damming) in order to decrease their natural methane emissions [68]. As atmospheric concentrations of CO_2_ and CH_4_ continue to rise, expanding monitoring capacity and process understanding of the C cycle, particularly in the data-poor tropics, is more urgent than ever.

## Data accessibility

*Guma Lagoon perennial wetland eddy-covariance and meteorological dataset*: Dataset available from the UK Environmental Information Data Centre. Helfter *et al.* (2021) https://doi.org/10.5285/d366ed40-af8c-42be-86f2-bb90b11a659e [69]. *Nxaraga seasonal floodplain eddy-covariance and meteorological dataset*: Dataset available from the UK Environmental Information Data Centre. Helfter *et al.* https://doi.org/10.5285/2170ebd0-7e6f-4871-97d9-1d42e210468f [70].

The data are provided in the electronic supplementary material [71].
